# Analysis of interrelated characteristics between ecosystem services and ecosystem health in the Guangdong–Hong Kong–Macao Greater Bay Area

**DOI:** 10.3389/fpls.2025.1668073

**Published:** 2025-09-29

**Authors:** Xiaojia Wang, Yushang Wang, Langxi Song, Seping Dai, Chuanfu Zang

**Affiliations:** ^1^ School of Geography, South China Normal University, Guangzhou, China; ^2^ Guangzhou Institute of Forestry and Landscape Architecture, Guangzhou, China; ^3^ Guangzhou Collaborative Innovation Center on Science-tech of Ecology and Landscape, Guangzhou, China

**Keywords:** ecosystem health, ecosystem services, interrelated characteristics, machine learning, the Guangdong–Hong Kong–Macao Greater Bay Area

## Abstract

Ecosystem health (EH) underpins the capacity of vegetation ecosystems to provide essential ecosystem services (ESs), which together are fundamental to regional sustainability. In regions undergoing rapid urbanization, the interrelationships between EH and ESs become increasingly complex, yet they remain largely unexplored in previous studies. This study integrates the VOR and InVEST models to quantify EH and four key ESs in the Guangdong–Hong Kong–Macao Greater Bay Area (GBA) from 2000 to 2020 and further analyzes their interrelationships using a bivariate spatial autocorrelation model and the XGBoost-SHAP approach. The results indicate that: (1) From 2000 to 2020, low-value areas of most ESs and EH expanded, regions of EH deterioration accounted for 71.75% of the study area, indicating the profound impact of rapid urbanization. (2) EH showed strong positive global spatial correlations with CS and NPP, but weak negative spatial correlations with FP and WY. (3) Interrelationships between ESs and EH can be divided into stable synergy type and dynamic trade-off type based on their differing ecological processes; climate factors can significantly impact the interrelationships primarily by affecting the dynamic trade-off type. This study integrates spatial analysis and machine learning approaches to examine the relationships between EH and ESs, thereby advancing the understanding of ecosystem states and functions and providing a theoretical basis for formulating ecological restoration targets.

## 1 Introduction

Ecosystems sustain human societies by delivering a wide range of benefits, which are commonly understood through the concept of ecosystem services (ES). Ecosystem services refer to the ecological attributes, processes, and functions that underpin human well-being, highlighting the ways in which functioning ecosystems contribute to people’s lives (Costanza et al., 1997). Urbanization profoundly alters natural ecosystems (Grimm et al., 2008), causing declines in ESs and significantly affecting the well-being of humans (Millennium Ecosystem Assessment, 2005). In this context, enhancing ESs to sustain the well-being of humans has become a significant issue guiding environmental conservation. However, ecosystems are complex systems influenced by multiple factors. To ensure the sustainable provision of ESs, ecosystem management must focus not only on ESs but also on their mechanisms of generation (Bennett et al., 2009), which corresponds to the condition of ecosystems. Therefore, the ultimate goal of ecosystem management is to ensure the provision of diverse ESs while maintaining optimal ecosystem health (EH) (Rapport et al., 1999). EH refers to the structural and functional integrity of an ecosystem, encompassing its ability to remain active, organized, and self-sustaining, while also demonstrating resilience to stress and disturbances (Costanza, 1992; Rapport et al., 1998). In addition to the provision of ESs, EH is also crucial, as it represents the concept of an ideal ecosystem. Therefore, a thorough investigation of the EH-ESs relationship in a specific region is crucial, which will serve as a scientific foundation for effectively promoting their integrated enhancement and coordinated improvement.

Regarding the interrelationship between ESs and EH, widespread consensus indicates that healthy ecosystems can provide ESs sustainably and steadily (Costanza, 2012; Hernández-Blanco et al., 2022). However, quantitative research reveals that relationships between the two may not always reflect strictly synergistic characteristics (Zhu and Cai, 2024). In regions undergoing rapid urbanization, where dramatic changes are occurring in the structure, function, and state of ecosystems, the complexity of interrelationship between EH and ESs may increase. Therefore, it is imperative to test the hypothesis that a healthy ecosystem produces high levels of ESs, especially in rapidly urbanizing areas. However, research on the integration of ESs and EH remains limited. Most related studies tend to examine EH and ESs separately. In studies on EH, scholars have primarily employed evaluation models such as PSR and VOR to quantitatively assess ecosystem health (Das et al., 2021; Li et al., 2024). Subsequent analyses often include investigations of driving factors and corresponding ecological zoning (Han et al., 2024; Li et al., 2025; Shao et al., 2025). Research on ecosystem services, which has become a central focus in contemporary ecology, mainly encompasses trade-off and synergy analyses based on the quantification of ecosystem services (Feng et al., 2021; Xue et al., 2023), cluster analysis (Dou et al., 2020; Xia et al., 2023; Su et al., 2024), and mechanism analysis of influencing factors (Liu et al., 2019; Jianying et al., 2020; Qi et al., 2020).

Existing studies that combine EH and ESs mainly link ESs to the assessment of EH (Peng et al., 2015; Pan et al., 2021; Yu et al., 2022; Huang et al., 2024), grounded in the theory that healthy ecosystems can provide abundant service functions. Some studies also take them as indicators to quantitatively measure ecological indices. For example, some scholars analyzed regional ecological risk by taking the square root of the product of ecosystem health and service function indicators in the Fen River basin (Wang et al., 2023a) and the Beijing-Tianjin-Hebei urban agglomeration (Kang et al., 2018). Other scholars have integrated EH and ESs with additional indicators and conducted comprehensive analysis of these indicators to measure ecological security in the Huaihe River Basin (Zhu and Cai, 2023). However, the above studies focus primarily on the integrated application of these two indicators rather than investigating the underlying relationship mechanisms between them. Given the fundamental differences between EH and ESs in conceptual frameworks and quantification methods, they are likely to exhibit distinct spatiotemporal patterns and response mechanisms. Therefore, it is necessary to understand their intrinsic relationships rather than simply integrating these indicators. In existing research on the interrelationship between EH and ESs, Liu et al. (2015), using correlation coefficient calculation methods, found a generally synergistic relationship in the forest areas of Northeast China. Similarly, Zhu et al. (2021), also employing correlation coefficient calculation methods, discovered that in the Pingjiang watershed, higher regional ecological quality is associated with lower food supply services, with the trade-off between NDVI and food supply being the most significant. In reality, ESs and EH are the results of interactions among numerous ecological variables, the relationship between them may exhibit complex non-linear characteristics. Correlation coefficients overlook the uncertainties introduced by these characteristics and fails to reflect the complex relationship. Therefore, further systematic analysis and research are needed to reveal the complex characteristics and mechanisms of the interrelationship between ESs and EH amid rapid urbanization.

As a prominent economic hub in China, the Guangdong–Hong Kong–Macao Greater Bay Area (GBA) is one of the four leading bay areas worldwide. By 2023, the total population of GBA had exceeded 86 million, and its economic output had surpassed 14 trillion yuan. Taking up a fraction of one percent of China’s land area, the GBA contributes to 1/9th of the national economic total, playing a pivotal role in the quality-driven growth of China (Wang et al., 2023b). The GBA boasts favorable natural geographic conditions, but rapid urbanization has stressed the natural ecosystems. Previous research has shown that the GBA is facing increasing environmental and resource pressure (Wu et al., 2021), and urban carbon emissions grew rapidly by 7% from 2000 to 2011 (Zhou et al., 2018). Therefore, the GBA is a typical example of changes in EH and ESs driven by rapid urbanization. Guided by the Green and Beautiful Guangdong Campaign, the GBA plays an essential role as a model for the development of bay areas in China and is positioned to be a pioneer for the Beautiful China Initiative, which makes ecological construction in the GBA extremely important. Consequently, enriching research on ecosystems in the GBA is important for providing the theoretical foundation for more scientifically informed ecological management practices. Therefore, this study integrates the InVEST model, VOR model, bivariate Moran’s I method and machine learning techniques to explore the interrelated characteristics between EH and ESs. This research aims to: (1) quantify EH and ESs, exploring their spatiotemporal distribution patterns; (2) examine the spatial interrelationships between EH and ESs; and (3) investigate the variable interrelated characteristics between EH and ESs. The findings will provide deeper insights into the interactions between EH and ESs, which will offer a valuable foundation for designing ecological restoration strategies in regions sharing comparable geographical characteristics.

## 2 Materials and methods

### 2.1 Study sites

**Figure 1:**
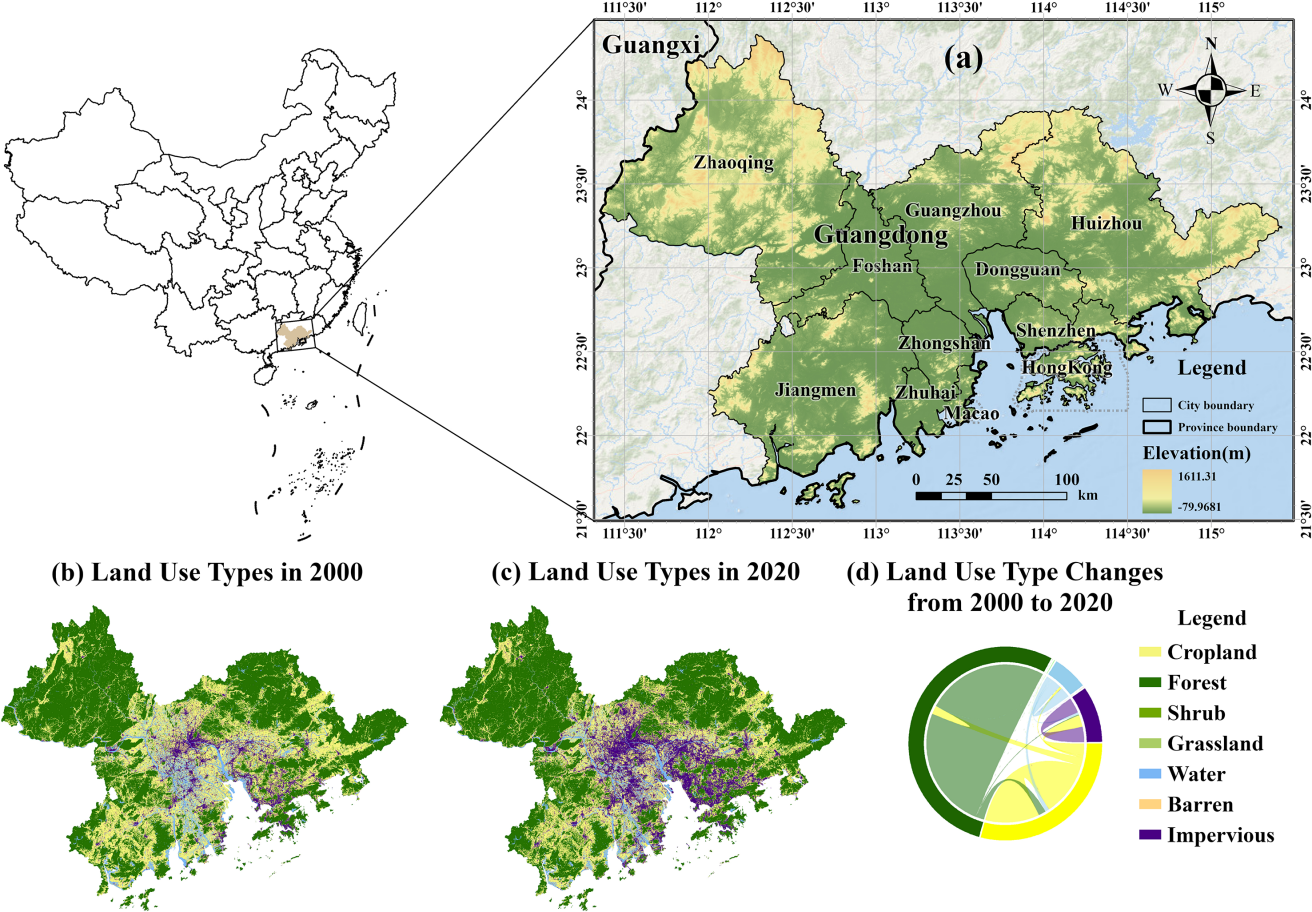
Geographical location **(a)** and land use types **(b-d)** of the Guangdong–Hong Kong–Macao Greater Bay Area.

The GBA spans an area of 5.6×10^4^ km^2^, covering coordinates from 21°25’ to 24°30’N and 111°12’ to 115°35’E (**Figure 1A**). It is characterized by an overlap of rainfall and temperature seasonality under a subtropical monsoon climate. It experiences warm and humid conditions year-round, which create favorable conditions for both heat and water. The mountains are concentrated in the northern part, while plains are predominantly located in the central region and along the coast. The dominant vegetation type is subtropical evergreen broadleaf forest. As a key economic hub of China, the GBA has undergone rapid urbanization in the past few decades, significantly transforming its surface landscape pattern (**Figures 1B-D**).

### 2.2 Data sources and methodology

Multivariate data were collected to quantify EH and ESs, and the data used are summarized in **Table 1**, where the first column lists the types of data employed, and the second and third columns provide the source of each dataset along with additional details such as spatial resolution and temporal coverage (**Table 1**).

**Table 1:** Data and their sources.

Data type	Data source	Data description
Land use	(Yang and Huang, 2021)	Five periods in 2000, 2005, 2010, 2015 and 2020; 30 m resolution
Digital Elevation Model (DEM)	Geospatial Data Cloud (https://www.gscloud.cn)	30m resolution
Meteorological data	China Meteorological Data Service Centre (http://data.cma.cn/en) and some district and county meteorological bureaus of Guangdong, Hunan, Guangxi, Fujian and Jiangxi Province	Daily scale data of meteorological stations from 2000 to 2020
Absolute depth to bedrock	International Soil Reference and Information Centre (ISRIC) (https://www.isric.org/)	250m resolution
Derived available soil water capacity (volumetric fraction) until wilting point	International Soil Reference and Information Centre (ISRIC) (https://www.isric.org/)	250m resolution
Residential and industrial	Open Street Map (https://www.openstreetmap.org/)	Shapefile data
Normalized Difference Vegetation Index	Chinese Academy of Science Discipline Data Center for Ecosystem (http://www.nesdc.org.cn/)	Five periods in 2000, 2005, 2010, 2015 and 2020; 30 m resolution
Net Primary Productivity	Analytical Insight of Earth (https://engine-aiearth.aliyun.com/#/portal/analysis)	Five periods in 2000, 2005, 2010, 2015 and 2020; 500 m resolution

To enhance the accuracy of spatial interpolation, we incorporated meteorological station data from both the GBA and its adjacent regions into calculation. In ArcGIS Pro 3.0.2 software, we obtained meteorological raster data by applying the Kriging spatial interpolation technique. We standardized all spatial datasets to the WGS 1984 UTM Zone 49N coordinate system based on the study area’s location.

### 2.3 Quantification of ecosystem health

We quantified EH using the VOR framework, which is considered to characterize ecosystem structure and function completely (Costanza, 2012; Das et al., 2021).

The VOR framework includes the assessment of ecosystem vitality, organization and resilience. We measured ecosystem vitality using the Normalized Difference Vegetation Index (NDVI), which is regarded as an effective way to evaluate the vitality (Box et al., 1989; Peng et al., 2015); Using the moving window method by Fragstats 4.2 software, we quantified ecosystem organization thorough three landscape metrics: Shannon’s Diversity Index (SHDI), Contagion Index (CONTAG), and Patch Cohesion Index (COHESION) (Frondoni et al., 2011; Peng et al., 2017). Using InVEST v3.14.1 software, we assessed ecosystem resilience through the Habitat Quality sub-module (Ye et al., 2013; Sharp et al., 2018; Chen et al., 2022). We used Equations 1–4 for the calculation:


(1) $$i_{rxy}=1-(\frac{d_{xy}}{d_{rmax}}),\text{if linear}$$

(2) $$i_{rxy}=\text{exp}\;(-(\frac{2.99}{d_{rmax}})d_{xy}),\text{if exponential}$$

(3) $$D_{xj}=\sum_{r=1}^{R}\sum_{y=1}^{Y_{r}}(\frac{W_{r}}{\sum_{r=1}^{R}W_{r}})r_{y}i_{rxy}\beta_{x}S_{jr}$$

(4) $$Q_{xj}=H_{j}(1-\frac{D_{xj}^{2}}{D_{xj}^{2}+{\text{k}}^{2}})$$

where $d_{xy}$ represents the linear distance between raster cells x and y ; $d_{rmax}$ denotes the maximum effective distance of threat r ’s reach across space; $i_{rxy}$ is the impact of the threat factor r originating from the cell x on the cell y ; $D_{xj}$ represents the habitat degradation risk index of the cell x in the habitat type j ; y is the cell under the threat factor r ; $r_{y}$ is used to determine if the cell y provides a source of threat factor r ; $W_{r}$ is the threat weight of the factor r ; $\beta_{x}$ denotes the accessibility of the threat factor of the cell x ; $S_{jr}$ represents the sensitivity coefficient of the land use type j to the factor r ; $Q_{xj}$ is the quality of habitat of the raster x in habitat type j ; and k is the half-saturation constant, often be set to 0.05.

The TOPSIS method is a widely used evaluation technique that identifies the best choice by calculating the distances between the data set and both the ideal and worst solutions (Hwang and Yoon, 1981). To reduce the subjectivity of weighting decisions, we combined the Entropy Weight method with the TOPSIS method for the quantification of EH (Lv et al., 2023; Wu et al., 2021).

### 2.4 Quantification of ecosystem services

We selected four ecosystem service indicators as representatives based on the context of the GBA, including carbon sequestration (CS), net primary productivity (NPP), food production (FP), and water yield (WY). (1) The dense population and advanced economic development in the GBA make the ecosystem’s provision of material and energy services crucial, and NPP reflects the material production capacity of the ecosystem (Nemani et al., 2003). (2) With the reduction in per capita farmland area over the past few decades (Zhang et al., 2021), research on FP is crucial for ensuring food security and understanding the effects of urbanization on FP. (3) As economic and population growth increases demand for clean water and hydroelectric resources, assessing WY helps optimize water resource management for sustainable development (Wang et al., 2021). (4) Carbon emissions are high in the GBA, while the superior vegetation conditions provide a basis for CS. Studying CS is important for achieving carbon neutrality (Zhou et al., 2018).

#### 2.4.1 Carbon sequestration

CS in a given area is determined by the size of four carbon pool: above-ground biomass, below-ground biomass, soil carbon, and dead organic matter. We used the Carbon Storage and Sequestration sub-module in InVEST v3.14.1 (Delphin et al., 2013; Sharp et al., 2018; Chu et al., 2019) to assess CS. We calculated the total carbon storage for each land use type using Equation 5:


(5) $$C_{totali}=\left(C_{abovei}+C_{belowi}+C_{soili}+C_{deadi}\right)\times A_{i}$$

where $A_{i}$ represents the area of land use type i ; $C_{abovei}$ is the above-ground carbon storage per unit area of land use type i ; $C_{belowi}$ is the below-ground carbon storage per unit area of land use type i ; $C_{soili}$ is soil carbon storage per unit area of land use type i ; $C_{deadi}$ is dead organic carbon storage per unit area of land use type i ; and $C_{totali}$ represents the total carbon storage of land use type i .

#### 2.4.2 Net primary productivity

NPP is the total amount of organic matter accumulated by vegetation per unit area and per unit time, reflecting the material supply capacity of the ecosystem (Nemani et al., 2003; Imhoff et al., 2004). We downloaded the MOD17A3HGF006 vegetation NPP dataset from the Analytical Insight of Earth Platform to assess NPP.

#### 2.4.3 Water yield

The Annual Water Yield sub-module in InVEST v3.14.1 is an estimation method using the water balance method (Redhead et al., 2016; Sharp et al., 2018), calculated WY by Equation 6:


(6) $$Y\left(x\right)=\left(1-\frac{AET\left(x\right)}{P\left(x\right)}\right)\cdot P\left(x\right)$$

where Y(x) represents the annual water yield (mm) of the raster cell x ; AET(x) is the annual actual evapotranspiration (mm) of the cell x ; P(x) is the annual precipitation (mm) of the cell x . We calculated evapotranspiration using the Penman-Monteith equation. The maximum root burial depth data required by the model was substituted with the absolute depth to bedrock data, while the plant available water content was calculated from available soil water capacity until wilting point data, which is recommended in the user’s guide in InVEST software (Sharp et al., 2018). Zhang’s coefficient is a constant that represents the seasonal characteristics of precipitation, we adjusted it for different years to ensure the model simulation results align with government water resource reports.

#### 2.4.4 Food production

Due to the significant linear correlation between grain production and NDVI (Chen et al., 2020), we combined the NDVI values with grain production statistics collected from the Statistical Yearbook to better quantify the value of food production (Huang et al., 2023). We computed FP using Equation 7:


(7) $$FP_{i}=G_{\text{sum }}\times \frac{{\bar{NDVI}}_{i}}{{\bar{NDVI}}_{\text{sum }}}$$

where $FP_{i}$ represents the food production value of raster cell i ; $G_{\text{sum }}$ represents the total regional production of crops; ${\bar{NDVI}}_{i}$ denotes the annual mean NDVI value of the cell whose land use type is farmland; and ${\bar{NDVI}}_{\text{sum }}$ represents the sum of NDVI values across all raster in the GBA. To achieve more realistic results, we performed calculations separately for each city based on the respective statistical data.

### 2.5 Analysis of the interrelated characteristics between EH and ESs

ESs and EH are two comprehensive indicators of ecosystems, and the relationship between the two is likely to be complex, as they result from the interaction of many environmental and ecological variables. Due to spatial interactions and diffusion, spatial autocorrelations can cause biases in the relationship between these indicators when using methods like ordinary least squares or geographically weighted regression model (Zhang et al., 2018). In contrast, the bivariate Moran’s I method effectively reflects the interrelationships of two variables spatially (Anselin, 1995; Xu et al., 2019), making it suitable for revealing bivariate spatial correlations. Thus, we use this method to reveal spatial interrelated characteristics between the indicators.

Besides research on spatial correlation features, combining methods that intuitively reveal the interrelated characteristics between variables may further enhance the understanding of their relationship. In recent years, machine learning models have gained popularity in geographic and ecological analysis due to their ability to effectively process multidimensional data and reveal nonlinear relationships between variables without predefined norms. The Extreme Gradient Boosting (XGBoost) model, which adds regularization rules to reduce the risk of overfitting, has significantly improved algorithm efficiency and accuracy, making it highly effective in ecological studies (Yang et al., 2021; Yuan et al., 2024). Additionally, SHAP (Shapley Additive Explanations), based on game theory and local interpretability, is a classic method used to explain the results of machine learning. The partial dependence plots generated by SHAP help in understanding the complex nonlinear interrelationships between explanatory variables and dependent variables (Lundberg et al., 2018). Based on this knowledge, we employed the XGBoost-SHAP model to further analyze the relationship between ESs and EH.

#### 2.5.1 Spatial interrelated characteristics

We examined the spatial interrelationships between EH and ESs via the GeoDa 1.18.0 software, employing both global and local spatial autocorrelation methods. We applied global Moran’s I to identify the overall spatial correlation between EH and ESs, with values ranging from -1 to 1. We used Equation 8 to calculate the global Moran's I.


(8) $$I_{global}^{ab}=\frac{n}{S_{0}}\frac{\sum_{i=1}^{n}\sum_{j=1}^{n}w_{ij}\left(X_{ai}-{\bar{X}}_{a}\right)\left(X_{bj}-{\bar{X}}_{b}\right)}{\sum_{i=1}^{n}{\left(X_{ai}-{\bar{X}}_{a}\right)}^{2}}$$

where n is the total number of spatial units; $X_{ai}$ and $X_{bj}$ are the observed values of variables a and b for spatial units i and j , respectively; ${\bar{X}}_{a}$ ​ and ${\bar{X}}_{b}$ ​ are their mean values; $w_{ij}$ ​ is the spatial weight describing the spatial relation between units i and j ; $S_{0}$ is the sum of spatial weights. $I_{global}^{ab}$ is the global bivariate Moran’s I index. A positive value suggests a positive spatial correlation, while a negative value suggests a negative spatial correlation. When $I_{global}^{ab}$ = 0, no spatial autocorrelation exists between the variables.

At the local level, we used the local Moran’s I (LISA) method to identify spatial agglomeration patterns between EH and ESs. We calculated local Moran's I using Equation 9:


(9) $$I_{i}^{ab}=z_{a,i}\sum_{j=1}^{n}w_{ij}z_{b,j}$$

where $z_{a,i}=\left(X_{ai}-{\bar{X}}_{a}\right)/\sigma_{a}$ and $z_{b,j}=\left(X_{bj}-{\bar{X}}_{b}\right)/\sigma_{b}$ are the standardized values of variables a and b ; $\sigma_{a}$ and $\sigma_{b}$ are their standard deviations; other symbols are as defined above. $I_{i}^{ab}$ is the local bivariate Moran’s I index. Based on the results of the bivariate Moran’s I analysis, we categorized the study area into five types: high-high type, indicating regions with high ESs and EH; low-low type, where both ESs and EH are low; high-low and low-high type, representing spatial heterogeneity; and non-significant type, indicating no obvious spatial agglomeration. In this study, we assessed the statistical significance of the results using a Z-test (P< 0.05).

#### 2.5.2 Variable interrelated characteristics

We implemented XGBoost-SHAP analysis using Python’s scikit-learn, XGBoost, and SHAP packages for modeling and visualization. Using ArcGIS Pro 3.0.2 software, we generated 1.0×10^5^ random points across the study area to extract ESs and EH data. Before modeling, we standardized all features using the StandardScaler to ensure comparability.

For constructing the XGBoost regression model, we utilized continuous EH values as the dependent variable, with multiple ecosystem service indicators serving as independent variables. The XGBoost algorithm builds predictive models through iterative addition of decision trees, and its core objective function is formulated as shown in Equation 10:


(10) $$ℒ=\sum_{i=1}^{n}l\left(y_{i},{\hat{y}}_{i}\right)+\sum_{k=1}^{K}\text{Ω}\left(f_{k}\right)$$

where n represents the total training samples, $y_{i}$ ​ and ${\hat{y}}_{i}$ ​ denote the observed and predicted values for sample i , respectively; $l\left(y_{i},{\hat{y}}_{i}\right)$ is the loss function; K indicates the total number of trees, $f_{k}$ ​ represents the k -th tree, and $\text{Ω}\left(f_{k}\right)$ is the regularization component that prevents overfitting.

We divided the dataset into training (80%) and validation (20%) sets. A grid search cross-validation (GridSearchCV) was performed to optimize the XGBoost hyperparameters, including maximum tree depth (3–6 nodes), learning rate (0.01-0.1), number of n estimators (100-300), min child weight (1, 3, 5 nodes) and subsample (0.8-1.0). We evaluated model performance by the coefficient of determination (R^2^). We employed Five-fold cross-validation to assess the model’s predictive accuracy and stability, ensuring robust performance across different data subsets.

For interpretability analysis, we employed SHAP methodology to quantify individual feature contributions to EH predictions. For a given observation i and feature j , the SHAP value is mathematically defined as shown in Equation 11:


(11) $$\phi_{j}\left(i\right)=\sum_{S\subseteq F\setminus \left\{j\right\}}\frac{\left|S\right|!\left(\left|F\right|-\left|S\right|-1\right)!}{\left|F\right|!}\left[f\left(S\cup \left\{j\right\}\right)-f\left(S\right)\right]$$

where F represents the complete feature space, S denotes any feature subset excluding j , and [f(S∪{j})−f(S)] quantifies the marginal contribution of feature j when added to subset S . The SHAP value $\phi_{j}\left(i\right)$ measures the contribution of feature j to the prediction for observation i . Feature importance ranking was determined by computing mean absolute SHAP values across all observations, providing insights into the relative influence of different ecosystem services on environmental health outcomes.

## 3 Results

### 3.1 Spatial-temporal variations of ESs and EH in the GBA from 2000 to 2020

**Figure 2:**
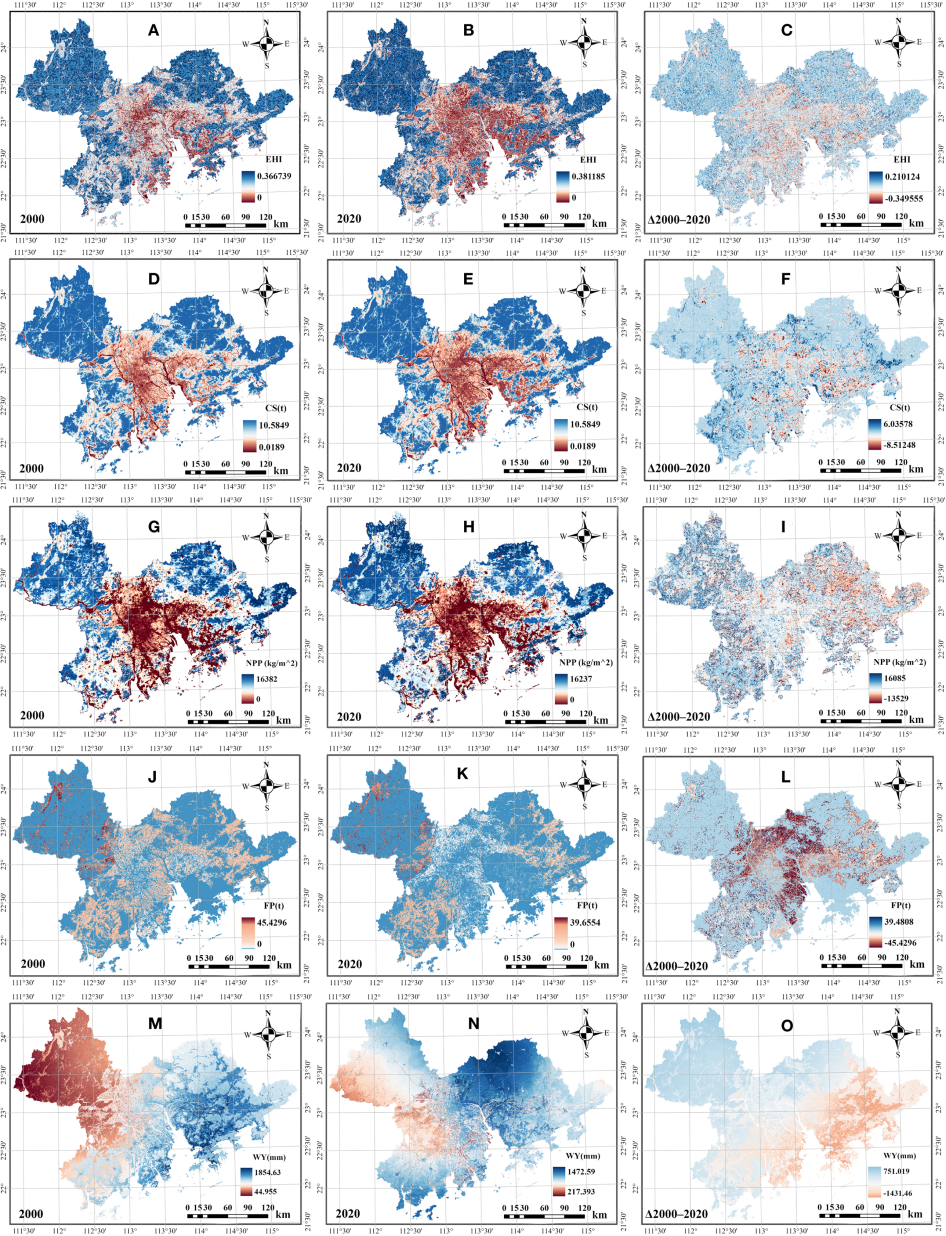
Quantification results and annual variations of EH **(A-C)** and ecosystem CS **(D-F)**, NPP **(G-I)**, FP **(J-L)**, and WY **(M-O)** service functions from 2000 to 2020.

Both ESs and EH in the GBA revealed prominent spatial heterogeneity, with the distribution of various indicators exhibiting spatial autocorrelation and significant differences in their spatial patterns (**Figure 2**). CS and NPP showed similar spatial distribution patterns to EH (**Figures 2A-I**), generally decreasing from the periphery to the center and from north to south. The highest values were concentrated in the northern parts of Zhaoqing, Guangzhou, and Huizhou, whereas the areas with lower values were predominantly concentrated in the core area of the GBA. Apart from areas such as Kowloon, Yau Tsim Mong, and Kwun Tong, where the values of EH, CS and NPP were lower, Hong Kong generally demonstrated exceptional EH and ESs conditions, emerging as concentrated zone of high ecological value within the central regions of the GBA. The high-value areas of FP demonstrated a more balanced spatial arrangement throughout the landscape with lower spatial autocorrelation (**Figures 2J-L**). Regions with high FP mostly concentrated in suburban areas, while regions with high WY formed a northeast-southwest corridor through the central of the GBA (**Figures 2M-O**).

**Figure 3:**
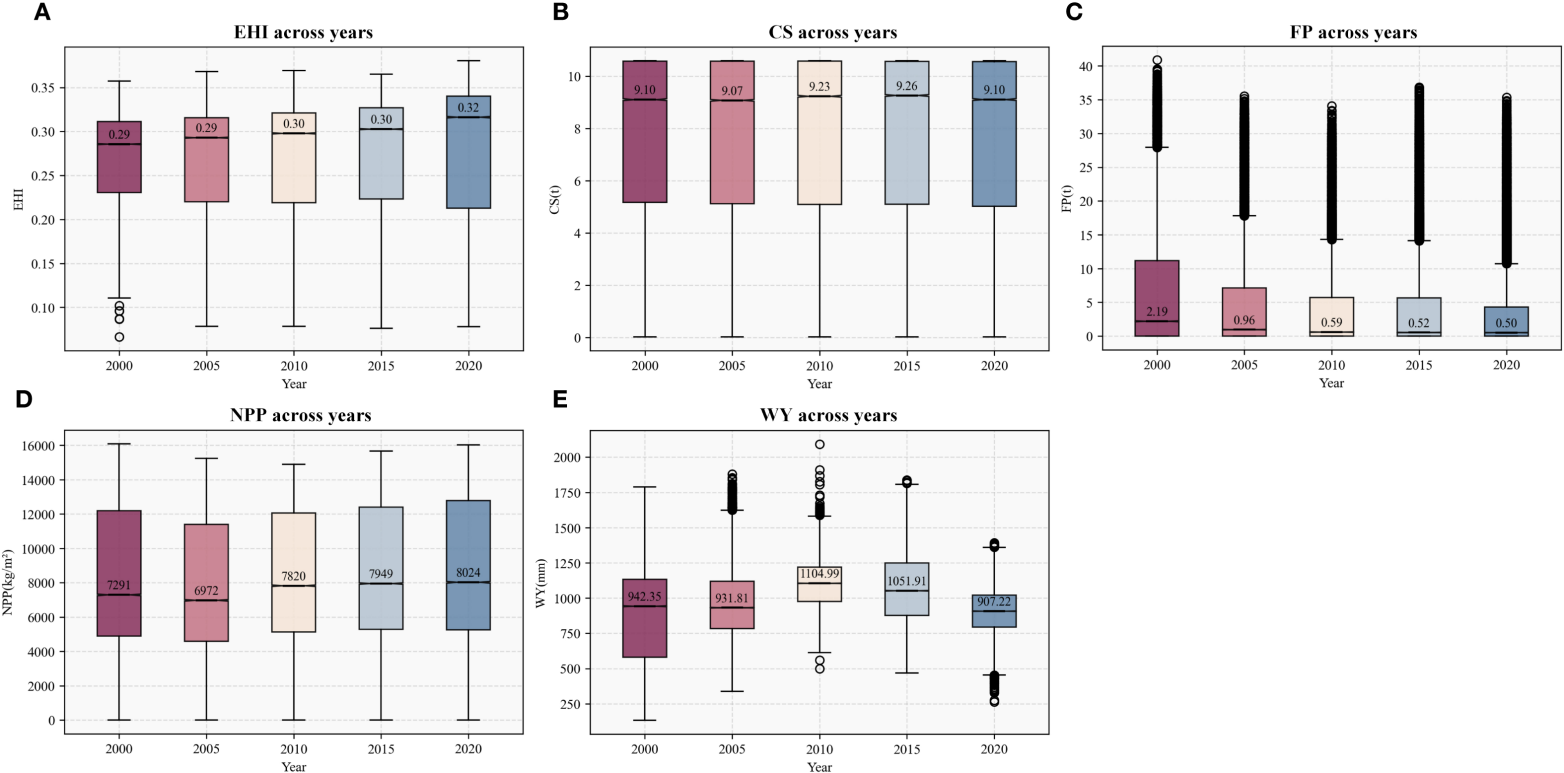
Boxplots of the values of EH **(A)** and ecosystem CS **(B)**, FP **(C)**, NPP **(D)**, and WY **(E)** service functions from 2000 to 2020.

Over the 20-year period, the median EHI showed a gradual increasing trend. The median EHI value rose from 0.29 in 2000 to 0.32 in 2020, while the dispersion of the data gradually increased over the 20-year period (**Figure 3A**). Spatially, low-value zones expanded, with 71.75% of the area experiencing EH degradation. However, values in most original high-value zones increased, resulting in an overall polarizing trend. The overall change trends of CS and NPP values were not pronounced (**Figures 3B, D**), but their spatial distribution patterns evolved similarly to EHI, with both indicators showing increased diffusion of low-value areas over time while surrounding areas exhibited relatively minor changes. FP decreased most significantly, with the median value per raster cell declining from 2.19t to 0.50t (**Figure 3C**). This sharp decrease in FP occurred primarily in the core areas of the GBA. Meanwhile, the data dispersion of FP also decreased significantly. WY exhibited the greatest magnitude of fluctuation, varying irregularly over time, with the median WY value changing by up to 197.77 mm (**Figure 3E**).

### 3.2 Spatial interrelated characteristics between ESs and EH in the GBA

**Figure 4:**
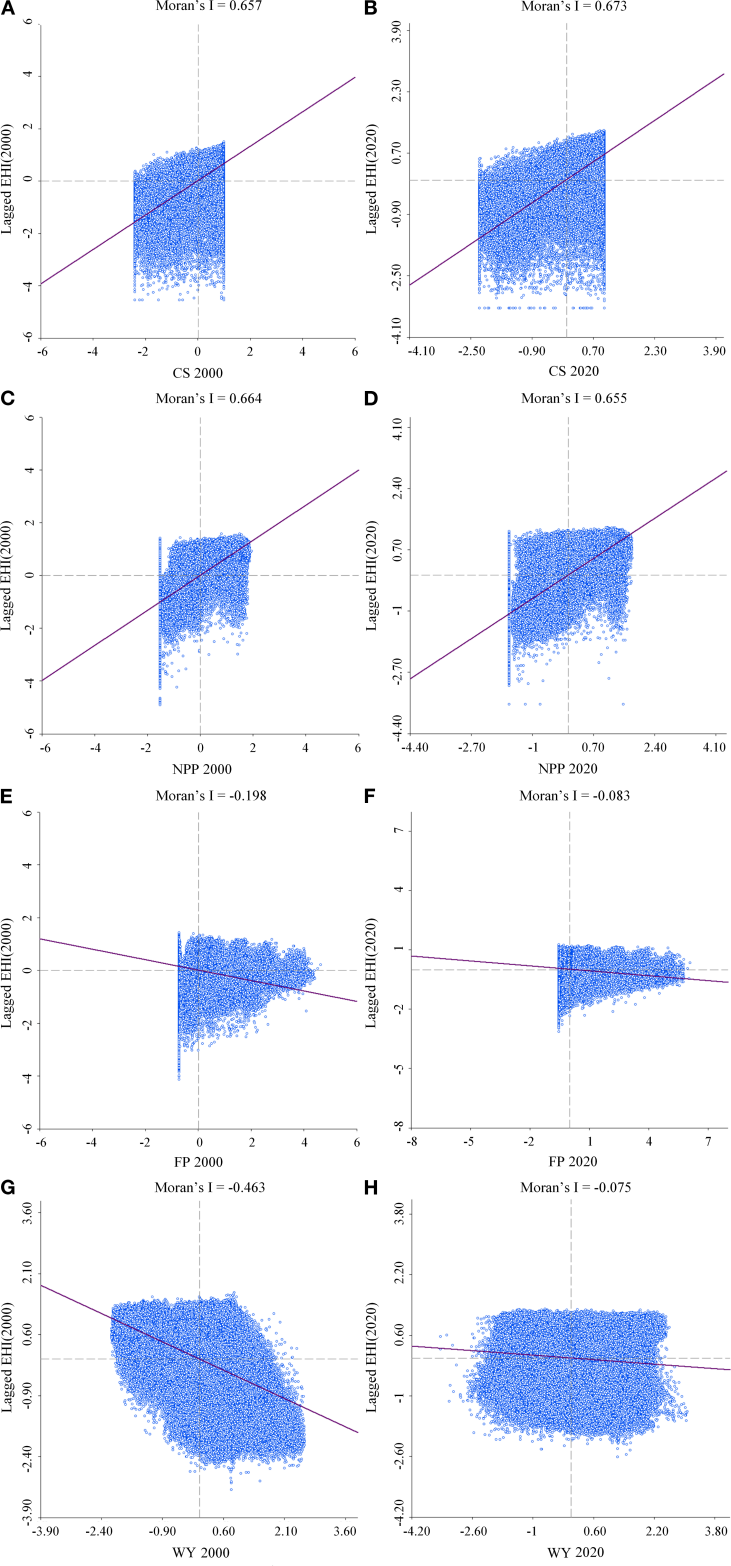
The bivariate Moran’s I scatter plots of EH and CS **(A, B)**, NPP **(C, D)**, FP **(E, F)**, and WY **(G, H)** from 2000 to 2020. The x-axis represents the standardized values of ESs, while the y-axis represents the spatially lagged EH values. Each graph includes a line of best fit and the corresponding Moran’s I value.

**Figure 5:**
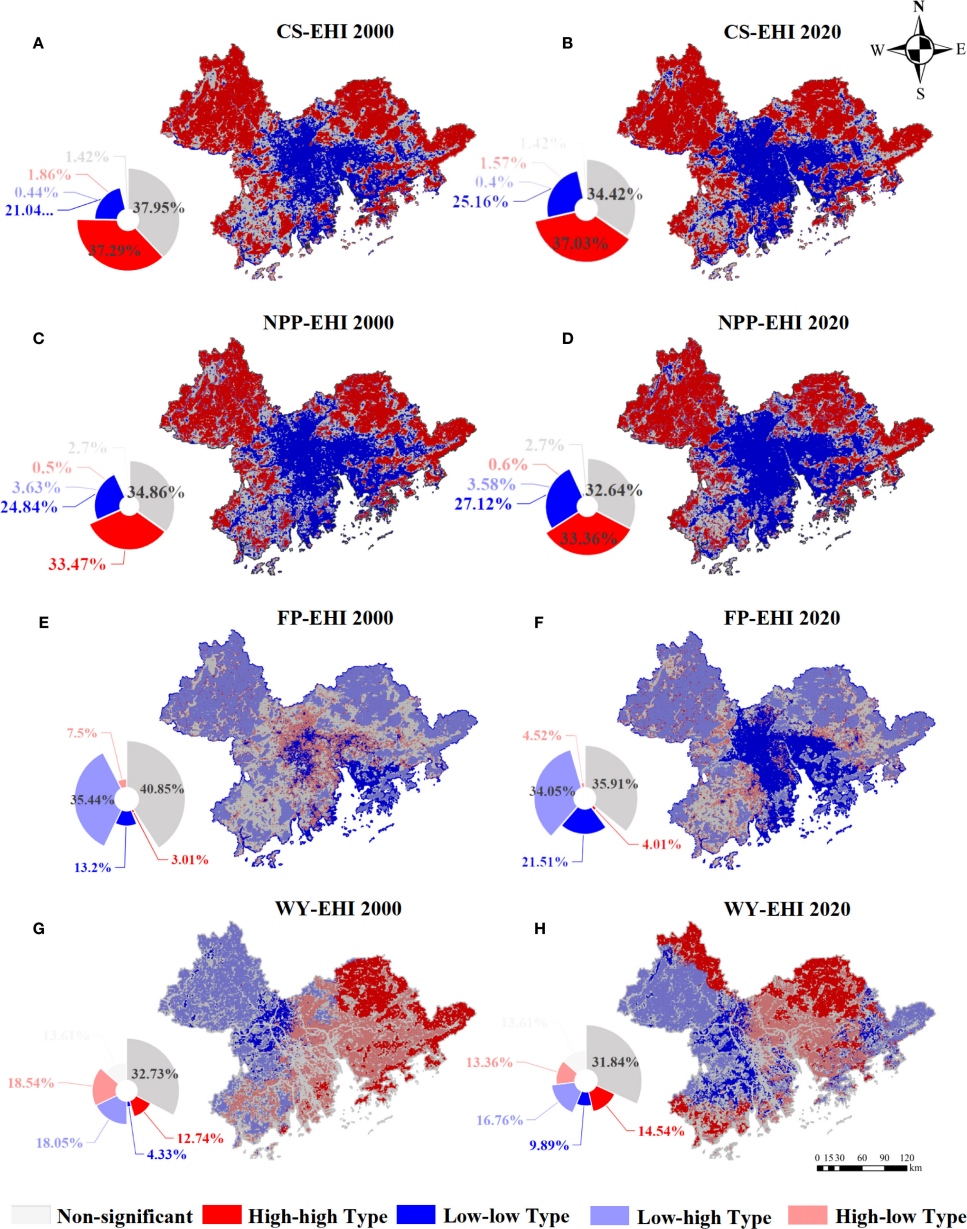
The bivariate LISA map of EH and CS **(A, B)**, NPP **(C, D)**, FP **(E, F)**, and WY **(G, H)** from 2000 to 2020. The pie chart represents the proportion of different clustering types in relation to the total area of the GBA.

Overall, the positive spatial correlations between ESs and EH predominated (**Figure 4**), with distinct spatial variations across different ES-EH combinations (**Figure 5**).

Global Moran’s I values exceeded 0.65 between EH and either CS or NPP, suggesting notable positive spatial correlation (P<0.05) (**Figures 4A-D**). The spatial clustering patterns between the two types of combination showed a high degree of overlap (**Figures 5A-D**), primarily characterized by the extensive distribution of high-high and low-low clusters. Over 30% of the area was occupied by high-high clusters, primarily located in the forested zones in the outskirts of the GBA. The low-low clusters covered over 20%, concentrated in the center of Guangzhou, Foshan, Shenzhen, and Dongguan.

Global Moran’s I values were negative for both EH-FP and EH-WY combinations (**Figures 4E-H**). The spatial clustering patterns for FP and EH were dominated by low-high clusters, which occupied more than 33% of the area and were located in the outskirts of the GBA (**Figures 5E, F**). The low-low clusters were predominantly located in the central regions of the GBA, surrounded by the low-high clusters. The spatial clustering pattern for WY and EH showed a more even distribution across types (**Figures 4G, H**), with the western part mainly exhibiting low-high and low-low clusters, and the eastern part showing high-low and high-high clusters. Temporally, the global Moran’s I between EH and either FP or WY decreased from 2000 to 2020. Low-low cluster areas expanded most notably in the EH-FP correlation pattern, with an increase of more than 8% in the GBA. The spatial interrelated characteristics between WY and EH varied dramatically with less discernible patterns.

### 3.3 Variable interrelated characteristics between ESs and EH in the GBA

The XGBoost model performance varied over time and among different ES and EH combinations (**Table 2**). The model of EH and CS achieved the highest accuracy with an average R² of 0.68, while the model of EH and NPP had an average R² of 0.53. The EH-WY model showed lower prediction accuracy with substantial temporal variability.

**Table 2:** Model fitting accuracy values (R^2^).

Years	R^2^ of different variables			
	CS	NPP	FP	WY
2000	0.7425	0.5711	0.3260	0.2817
2005	0.6589	0.5059	0.2262	0.1146
2010	0.6668	0.4980	0.2143	0.2980
2015	0.6622	0.4775	0.1835	0.2488
2020	0.7107	0.5245	0.2037	0.0469

**Figure 6:**
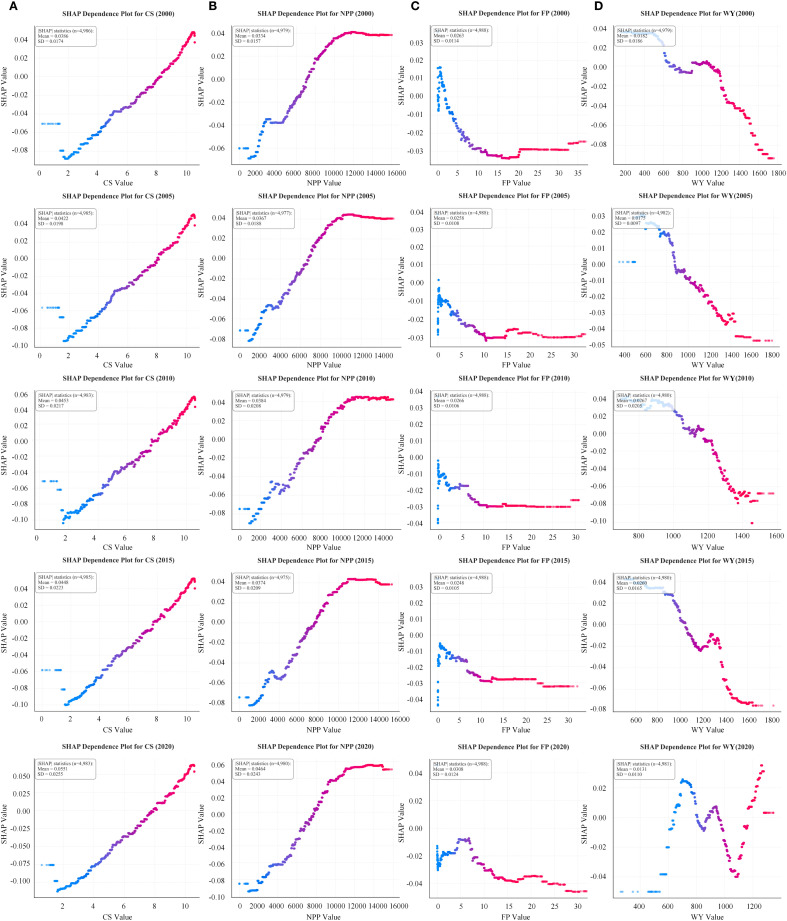
Interrelated characteristics between EH and CS **(A)**, NPP **(B)**, FP **(C)**, and WY **(D)** indicators from 2000 to 2020.

Results of the XGBoost-SHAP model indicated that CS and EH showed a positive relationship close to linear (**Figure 6A**). The relationship between NPP and EH showed a clear threshold effect, with SHAP values stabilizing and even slightly declining when NPP reaches around 10,000 kg/m² (**Figure 6B**). The relationship between FP and EH initially exhibited a positive trend before shifting to a negative trend at lower values. When FP exceeded 10 tons, the EH prediction curve flattened. From 2000 to 2020, the curve’s peak point shifted rightward (**Figure 6C**). The relationship between WY and EH demonstrated a negative correlation because the prediction curves showed notable variation across years (**Figure 6D**).

## 4 Discussion

Scholarly consensus suggests that healthy ecosystems can provide diverse ESs (Costanza, 2012). However, despite their conceptual relationship, the empirical evidence for specific relationships between EH and ESs within defined spatial-temporal contexts remains limited. To address this gap, our study leverages a combination of bivariate spatial autocorrelation modeling and the XGBoost-SHAP approach to quantitatively examine the interrelated characteristics between EH and ESs in the GBA, while the further understanding of the mechanisms behind these relationships and the comprehensive interaction between EH and ESs requires analysis of ecosystem processes.

### 4.1 Stable synergistic interrelated pattern between ESs and EH

EH showed similar spatial aggregation patterns with CS and NPP, characterized by significant positive correlations. Core areas with intense human activity experienced rapid urbanization over the past two decades, leading to declines in both EH and ESs and resulting in low-value and deteriorated zones (Gordon et al., 2009). In contrast, the outskirts of the GBA, less affected by urbanization, exhibited strong ecosystem vitality, organization, and resilience, supporting more stable material cycling and energy flow, and allowing rapid recovery from disturbances, thereby promoting CS and NPP (Chen et al., 2018; Reader et al., 2023). The gradual increase of the EH in surrounding areas can be attributed to ecological restoration projects in nearby mountains (Feng et al., 2021), plant growth promoted by climate change (Friend et al., 2013), or the self-recovery capacity of the ecosystem (Liu et al., 2023); all of these factors may enhance EH, CS, and NPP and reinforce their synergistic relationship.

At the process level, healthy vegetation ecosystems maintain high NPP and CS through photosynthesis, during which carbon is fixed and transformed into organic matter (Johnson, 2016; Walther et al., 2019; Smith et al., 2024). This establishes the coupling between CS and NPP and contributes to the stability of the ecosystem. Similar synergistic patterns have been observed elsewhere; for instance, Zhang et al. (2022) reported a correlation coefficient of 0.81 between habitat quality and carbon storage in the Chengdu–Chongqing Urban Agglomeration, and Huang et al. (2023) found a strong synergy between habitat quality and NPP in the Wujiang Basin. Urban land encroachment disrupts these ecological processes, reducing associated service functions, which has been widely observed in previous studies (Wu et al., 2024; Yushanjiang et al., 2024). Methodologically, previous studies often relied on correlation coefficients to assess overall relationships between ecosystem indicators, while more detailed analyses revealed spatial patterns of trade-offs and synergies (Xue et al., 2023). In this study, SHAP analysis further elucidated specific associations between EH and ESs. Notably, we identified a threshold effect in the NPP–EH relationship: areas with high NPP often corresponded to plantations around the GBA. Despite rapid material production, these plantations exhibit simplified ecosystem structures and are less capable of maintaining ecosystem health and multiple services (Dislich et al., 2017; Guillaume et al., 2018).

### 4.2 Dynamic trade-off interrelated pattern between ESs and EH

FP and WY exhibited more complex non-linear relationships with EH. EH increased initially with FP, but after a certain point, it decreased and then stabilized. FP is mostly enhanced by the use of pesticides, herbicides, and monoculture (Reader et al., 2023); these agricultural activities have resulted in simpler ecosystem structures and lower biodiversity, despite crop growth directly corresponding to increased ecosystem vitality on cropland. This dual effect explains the observed non-linear relationship between FP and EH. Temporally, from 2000 to 2020, the peak of the SHAP curve describing the relationship between FP and EH shifted to the right. The GBA used to be a major grain-producing region in Guangdong Province; however, rapid urbanization has directly consumed farmland, leading to decreased grain production (Wang et al., 2019). Consequently, regions with low EH and high FP shifted to low-low clusters, weakening the trade-off intensity between them, possibly contributing to the shift of the peak of the curve. Previous studies have indicated negative correlations between FP and other services; for example, Xia et al. (2023) revealed this pattern while also noting an expansion of synergy areas over time using a geographically weighted regression model. In contrast, our analysis found relatively few FP–EH trade-off zones, likely because the highly urbanized GBA contains extensive areas with simultaneously low EH and FP, weakening the observed trade-offs.

The interrelationship between EH and WY was not dominated by any single clustering type, and WY increased overall with noticeable fluctuations as EH decreased. The irregular clustering patterns and sharp fluctuations observed in the curves can largely be attributed to climatic variability, particularly precipitation. As an external driver, precipitation exhibits high uncertainty and weak correlation with surface conditions (Runting et al., 2017). Since WY is strongly controlled by precipitation, it exhibited greater variability than the relatively stable EH, leading to unstable interrelationship patterns. High–low clustering of WY and EH was mainly concentrated on the impervious surfaces at the urban core of the GBA, where rising WY due to blocked infiltration was accompanied by low EH. By contrast, forested areas showed higher EH but lower WY because of intense transpiration (Sun et al., 2006). Previous studies have reported higher WY in mountainous forests and lower WY in densely populated areas (Darvishi et al., 2022; Mo et al., 2023). The WY distribution in the GBA differs from these patterns, likely due to its monsoon-influenced coastal climate and extensive impervious surfaces typical of a large urban agglomeration. Regarding trade-offs between WY and other ecological indicators, prior studies have documented similar phenomena. For example, Liu et al. (2020) quantified runoff coefficients in Southwest China, revealing trade-offs between WY and carbon sequestration, while Zhang et al. (2022) found that in the Chengdu–Chongqing urban agglomeration, WY and habitat quality were largely dominated by trade-offs, with negative synergies exceeding positive ones. In contrast, our results show that in the GBA, high–high EH–WY clusters are more extensive than low–low clusters, likely reflecting abundant precipitation supporting higher WY. Methodologically, Zhang et al. focused on temporal trends, capturing WY declines during forest restoration, whereas our study emphasizes spatial patterns, revealing the coexistence of different ecological indicators across the GBA. Notably, SHAP analysis further identified the non-linear characteristics of the EH–WY relationship, highlighting its complexity under the combined influence of climate variability and human activities.

### 4.3 Hierarchical interrelated patterns between ESs and EH

The above analysis reveals that the interrelated patterns between ESs and EH vary depending on the type of ESs. This difference arises from the varying dependencies of ESs on ecosystem structures and processes.

CS and NPP are strongly correlated with ecosystem photosynthetic processes and serve as crucial indicators reflecting fundamental ecosystem metabolic processes. Their stable synergistic relationship with EH fully demonstrates the intrinsic resilience characteristics of ecosystems (Holling, 1973). In contrast, FP and WY are driven mainly by external factors such as human activities and climate change, reflecting ecosystems’ sensitive responses to external disturbances. Their dynamic trade-off relationship with EH profoundly reflects the adaptive adjustment processes of ecosystems within disturbance-recovery cycles (White and Pickett, 1985). Notably, the intensity of ecosystem responses to external disturbances tends to gradually diminish as ecosystem health status and fundamental supporting service functions improve. For instance, photosynthetic processes enhance the ecosystems’ buffering capacity against climate change by efficiently absorbing carbon dioxide and promoting long-term carbon storage. Under sustained moderate disturbance conditions, ecosystems can continuously evolve and enhance their stability (Holling, 1973; Walker et al., 2004). However, rapid urbanization has disrupted the inherent self-regulation thresholds of ecosystems (Muradian, 2001), making the differentiation mechanism of stability-dynamics interrelationship patterns more pronounced. This differentiation primarily stems from profound changes in land surface environmental characteristics induced by urbanization. These modifications not only amplify the fluctuation magnitude of key dynamic factors—such as temperature and precipitation—but also significantly intensify both the impact intensity and influence depth of these factors on corresponding ecosystem services.

The identification of hierarchical interrelated patterns represents an advancement beyond the traditional binary cognition of synergy-trade-off relationships between ecosystem attributes. By revealing the intrinsic hierarchical nature of ecosystem service-health relationships under rapid urbanization, this study provides empirical evidence. It offers novel insights into the classical theory that healthy ecosystems can sustainably provide abundant ESs. The stable synergistic characteristics confirm that the healthy ecosystems can maintain fundamental ecological processes. Meanwhile, the dynamic trade-off characteristics indicate that the supporting role of EH in supporting ESs is not a simple linear facilitation, but rather exhibits hierarchical differences based on the characteristics of ecological processes.

### 4.4 Limitations and future perspectives

This study investigates the interrelated characteristics between ESs and EH, advancing the understanding of their interactions. However, there remain certain limitations. Firstly, methodological constraints, including parameter dependencies in the InVEST model and subjective evaluations in the VOR model, may introduce quantification biases that potentially compromise regression fitting accuracy. Secondly, the types of ESs examined in this study are limited, necessitating future comprehensive investigations across a broader spectrum of ecosystem service types. Finally, while this study reveals the interrelated characteristics among indicators and provides reasonable interpretations under specific conditions, the underlying causal mechanisms require further investigation. This could be achieved through multi-scale and spatial-temporal quantitative analyses to provide more robust evidence for understanding the mechanistic interactions between ecosystem health and ecosystem services.

## 5 Conclusions

This study employs a comprehensive approach combining the InVEST model, VOR model, bivariate spatial autocorrelation analysis, and XGBoost-SHAP model to systematically investigate the interrelationships between ESs and EH in the Guangdong–Hong Kong–Macao Greater Bay Area. The findings reveal three key insights:

1. Over the past 20 years, values of EH and FP have decreased in the urban expansion areas of the GBA, while EH has improved in the peripheral zones. WY achieved high values in built-up areas, and the overall distribution pattern shows a spatial configuration of high values in the east and low values in the west. Rapid urbanization has significantly influenced the spatiotemporal evolution of EH and ESs in the GBA.
2. CS and NPP, as indicators closely linked to fundamental ecosystem metabolism, exhibit a stable synergistic relationship with EH. In the peripheral regions of the GBA, EH and corresponding service functions may be synergistically enhanced, requiring attention to the protection of natural forest lands.
3. FP and WY respond dramatically to human activities and climate change factors, showing a dynamic trade-off relationship with EH. Rapid urbanization has made ecosystems more sensitive to external disturbances, thereby intensifying the trade-off effects between EH and ESs.

By quantifying the relationship characteristics between ecosystem health and service functions, this research, based on the analysis of ecosystem structural and process characteristics, explores how ecosystems respond to external disturbances such as climate change in urban contexts, and further reveals the hierarchical differences in the relationship characteristics between EH and ESs as well as the impact of rapid urbanization on their relationship. This validates existing theories while providing new insights into their real-time manifestations during rapid urbanization. Furthermore, the results offer a theoretical basis for ecosystem management and planning in regions worldwide with similar natural geographic characteristics and urbanization development traits.

## Data Availability

The datasets presented in this article are not readily available because Dataset available on request from the authors. The raw data supporting the conclusions of this article will be made available by the authors on request. Requests to access the datasets should be directed to chuanfuzang@163.com.
